# Comparative structural insight into the unidirectional catalysis of ornithine carbamoyltransferases from *Psychrobacter* sp. PAMC 21119

**DOI:** 10.1371/journal.pone.0274019

**Published:** 2022-09-23

**Authors:** Hackwon Do, Dieu Linh Nguyen, Chang Woo Lee, Min Ju Lee, Hoejung Oh, Jisub Hwang, Se Jong Han, Sung Gu Lee, Jun Hyuck Lee

**Affiliations:** 1 Research Unit of Cryogenic Novel Material, Korea Polar Research Institute, Incheon, Republic of Korea; 2 Department of Polar Sciences, University of Science and Technology, Incheon, Republic of Korea; 3 Division of Life Sciences, Korea Polar Research Institute, Incheon, Republic of Korea; Universitetet i Bergen, NORWAY

## Abstract

Ornithine carbamoyltransferases (OTCs) are involved in the arginine deiminase (ADI) pathway and in arginine biosynthesis. Two OTCs in a pair are named catalytic OTC (cOTC) and anabolic OTC (aOTC). The cOTC is responsible for catalyzing the third step of the ADI pathway to catabolize citrulline into carbamoyl phosphate (CP), as well as ornithine, and displays CP cooperativity. In contrast, aOTC catalyzes the biosynthesis of citrulline from CP and ornithine *in vivo* and is thus involved in arginine biosynthesis. Structural and biochemical analyses were employed to investigate the CP cooperativity and unidirectional function of two sequentially similar OTCs (32.4% identity) named *Ps*_cOTC and *Ps*_aOTC from *Psychrobacter* sp. PAMC 21119. Comparison of the trimeric structure of these two OTCs indicated that the 80s loop of *Ps*_cOTC has a unique conformation that may influence cooperativity by connecting the CP binding site and the center of the trimer. The corresponding 80s loop region of in *Ps*_aOTC was neither close to the CP binding site nor connected to the trimer center. In addition, results from the thermal shift assay indicate that each OTC prefers the substrate for the unidirectional process. The active site exhibited a blocked binding site for CP in the *Ps*_cOTC structure, whereas residues at the active site in *Ps*_aOTC established a binding site to facilitate CP binding. Our data provide novel insights into the unidirectional catalysis of OTCs and cooperativity, which are distinguishable features of two metabolically specialized proteins.

## Introduction

Ornithine carbamoyltransferases (OTCs; EC2.1.3.3) are the enzymes involved in the arginine deiminase (ADI) pathway and arginine biosynthesis [1]. A pair of distinct OTCs are named the catabolic OTC (cOTC) and anabolic OTC (aOTC). The cOTC catalyzes the phosphorolysis of citrulline, yielding ornithine (ORN) and carbamoyl phosphate (CP), which serve to generate ATP from ADP via carbamate kinase in the ADI pathway. Although the anabolic reaction of cOTC can be measured *in vitro*, cOTC does not carry out the biosynthetic reaction (anabolism) *in vivo* owing to a poor affinity and cooperativity toward CP [2–5]. The aOTC is involved in the sixth step of the arginine biosynthetic pathway, wherein it reversely catalyzes the formation of citrulline and phosphate from ORN and CP by transferring the carbamoyl group with no allosteric kinetics [1, 6]. As these two OTCs have similar reactions but unidirectional catalysis, the tertiary structure of both proteins is highly similar.

Several structures of cOTCs were determined using X-ray crystallography, and their mechanisms have been discussed [7–11]. The cOTC from *Pseudomonas aeruginosa* (*Pae* cOTC) forms a dodecamer composed of four trimers in a tetrahedral manner, and was shown to exhibit allosteric activity [10]. AMP and other nucleoside monophosphates allosterically stimulate the enzymes, whereas the polyamines inhibit the allosteric reaction [4]. It was suggested that the homo-dodecameric state of the protein could be correlated with the homeotropic CP cooperativity and the thermal stability [12]. Mutational analysis of *Pae* cOTC confirmed that the C-terminal region contains an important element for oligomerization formation. However, the cOTCs from *Lactobacillus hilgardii* (*Lhi* cOTC, UniProt: Q8G998) and *Halobacterium halobium* (UniProt: Q48296) [13] are hexamers, thus indicating that the dodecameric structure of cOTC may not be a cOTC-specific structural feature explaining its catabolic activity [12].

Anabolic OTCs (aOTCs) also show diverse oligomerization states. The aOTC from the thermophilic bacterium *Pyrococcus*. *furiosus* (*Pfu* OTC, UniProt: Q51742) [14] exhibits a dodecameric assembly, whereas the aOTCs from *Mycobacterium tuberculosis* (*Mtb* aOTC, UniProt: P9WIT9) and *Escherichia*.*coli* (*E*. *coli* aOTC, UniProt: P04391) form trimers. In addition, the aOTC from the psychrophilic bacterium *Moritella abyssi* (OTCase_Mab_) exhibited a dodecamer state at high salt concentrations, which is not related to any allosteric properties [15].

Besides the oligomerization of OTCs, the biochemical characteristics, and structural comparisons have been conducted for detailed mechanisms [7, 16, 17]. For example, a structural comparison of apo *Mtb* aOTC and a complex with CP and l-norvaline (NVA) revealed that the SMG loop and 80s loops are the main regions affected by dual substrate binding [17]. A similar conformation change was found in human aOTC [16]. Furthermore, a comparison of *Pae* cOTC (apo) and *E*.*coli* OTC complexed with the bisubstrate analog N-(phosphonacetyl)-L-ornithine also confirmed domain closure and conformational change in the SMG loop [7]. However, a structure-based comparison of the unidirectional catalytic processes of the two enzymes, the distinguishable characteristics between cOTC and aOTC, have not been elucidated owing to a lack of studies on aOTC and cOTC from the same species. Here, we determined the *Ps*_cOTC and *Ps*_aOTC structures from the psychrophilic bacteria *Psychrobacter* sp. PAMC 21119 at 2.6 and 2.2 Å and compared them in respect to the substrate preferences for kinetic processes and cooperativity.

## Results and discussion

### Genetic composition and sequence analysis of *Ps_*cOTC and *Ps_*aOTC

**Fig 1:**
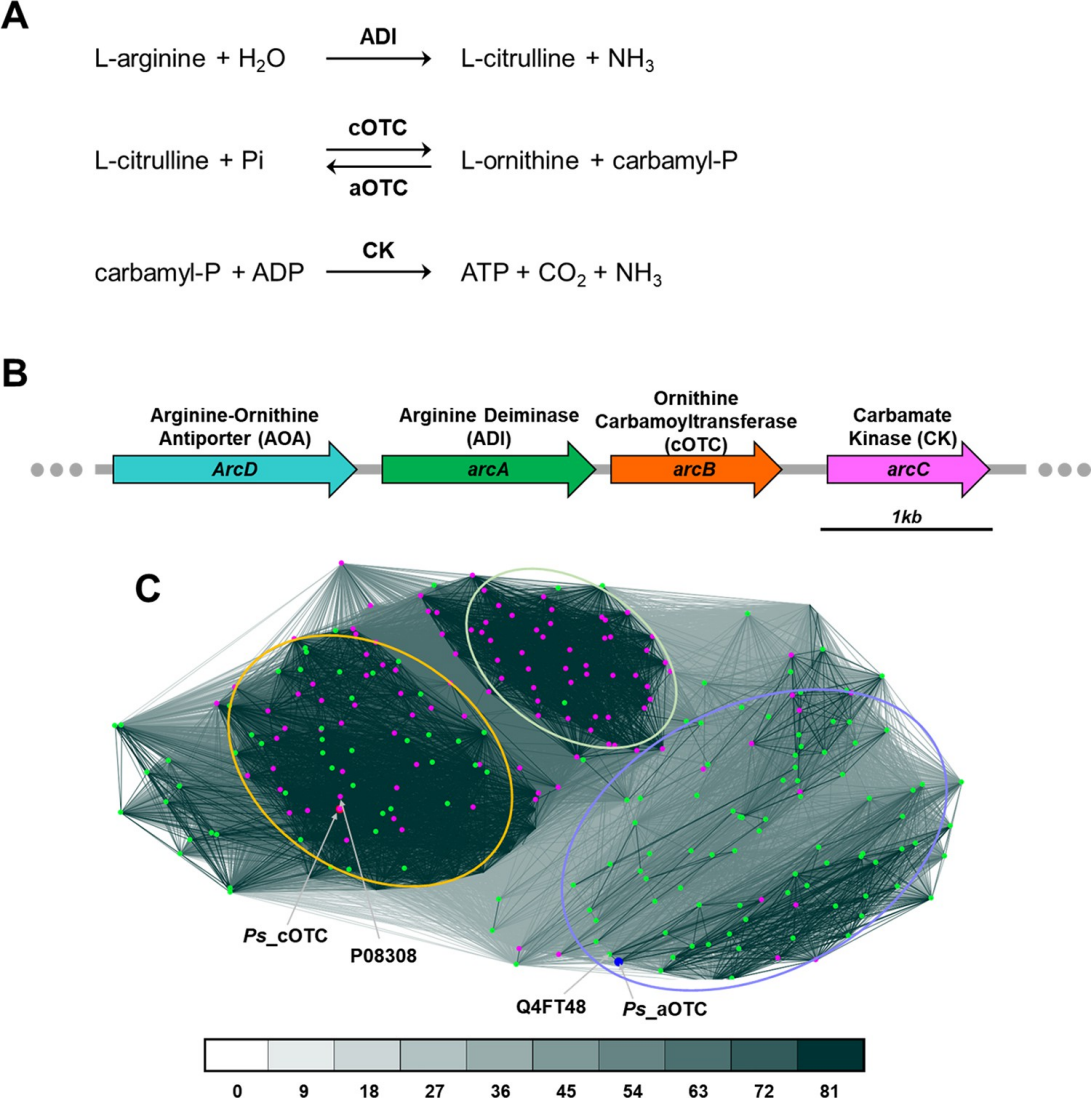
The catabolic OTC (cOTC) and anabolic OTC (aOTC) from *Psychrobacter* sp. PAMC 21119. (A) Schematic representation of the ADI pathway and the reaction catalyzed by the OTCs. (B) Schematic representation of gene organization within the ADI system of the PAMC 21119 strain. (C) Clustering analysis of OTCs searched using ProtBLAST/PSI-BLAST from the PBD and Uniprot_sport databases. The three clusters are indicated with light green, purple-blue, and orange circles. The genes annotated with *arcB* or *argF* are indicated with green and purple dots. *Ps*_cOTC and *Ps*_aOTC are indicated with red and blue dots. Darker and shorter connecting lines indicate higher sequence similarity. Connections with P-values higher than 1e-x are drawn in the corresponding color (x = number below).

Bioinformatic analysis identified a pair of OTCs from the genomic DNA of the *Psychrobacter* sp. PAMC 21119. The catabolic *Ps_*cOTC encoded by the *arcB* gene was involved in the operon of the ADI system (Fig 1A and 1B). The ADI system consists of four genes named *arcD*, *arcA*, *arcB*, and *arcC* that are transcribed in the same direction and separated by small intergenic regions. The *arcB* is located between *arcA* and *arC*. The *argF* (gene locus_tag: RH96_RS05760) encoding anabolic OTCase (*Ps_*aOTC) is remotely located.

Clustering analysis was performed using protein sequences with homologues obtained by BLAST against the Protein Data Bank (PDB) and UniProt databases. Sequence-based clustering revealed that the OTCs could be divided into three groups: one mixed OTC group and two distinguishable cOTC and aOTC groups, with several exceptions. The mixed group was formed mainly of gram-negative bacteria, such as OTCs from *E*. *coli*, *P*. *aeruginosa*, *Burkholderia pseudomallei*, *Vibrio parahaemolyticus*, and *Neisseria meningitides*. The cOTC group could be characterized by the tight clusters, which were mainly from gram-positive bacteria, such as *Streptococcus pyogenes*, *Streptococcus pneumonia*, and *Staphylococcus aureus* (marked with light green in Fig 1C). Considering that the shorter and darker lines in Fig 1C illustrate a high degree of sequence similarity, the aOTCs as the third cluster exhibited sequential diversity from various bacteria. The *Ps_*cOTC belongs to the mixed OTC group, whereas the *Ps_*aOTC belongs to the aOTC group. The closest OTC to the *Ps_*cOTC was the *Pae*_OTC from *P*. *aeruginosa* (UniProt code: P08308), while the closest OTC to the *Ps_*aOTC was the OTC from *Psychrobacter arcticus* (strain DSM 17307) (UniProt code: Q4FT48) (S1 Table).

Consistent with this result, multiple sequence alignment of *Ps_*cOTC and *Ps_*aOTC with their orthologues, which have been deposited in the PDB, indicated that the OTCs could be classified into two groups (Fig 4B). *Ps_*cOTC and *Ps_*aOTC could be included in each group. The *Ps_*cOTC group contains *Pae*_cOTC from *P*. *aeruginosa*, *Vvu OTC* from *Vibrio vulnificus*, *Lhi*_cOTC from *Lentilactobacillus hilgardii*, and *Stm*_OTC from *Salmonella typhimurium* (S3 Table). The other group contains *Ps_*aOTC, OTCs from *Burkholderia* sp., *Bme*_OTC from *Brucella melitensis*, *Pfu*_aOTC from *P*. *furiosus*, *Tma*_OTC from *Thermotoga maritima*, and *Mtb* aOCT from *M*. *tuberculosis* (S4 Table). Notably, the anabolic *E*. *coli*_aOTC has the sequential characteristics of the cOTC group. Conclusively, *Ps_*cOTC and *Ps_*aOTC are remotely separated by a relatively low sequential similarity (32.4% identity and 52.2% similarity) and belonged to different clustering groups. In addition, OTCs from various species can be grouped by sequences and the probable structural features can be correlated with the biochemical characteristics of cOTC and aOTC.

### Ligand binding mechanism of OTCs

**Fig 2:**
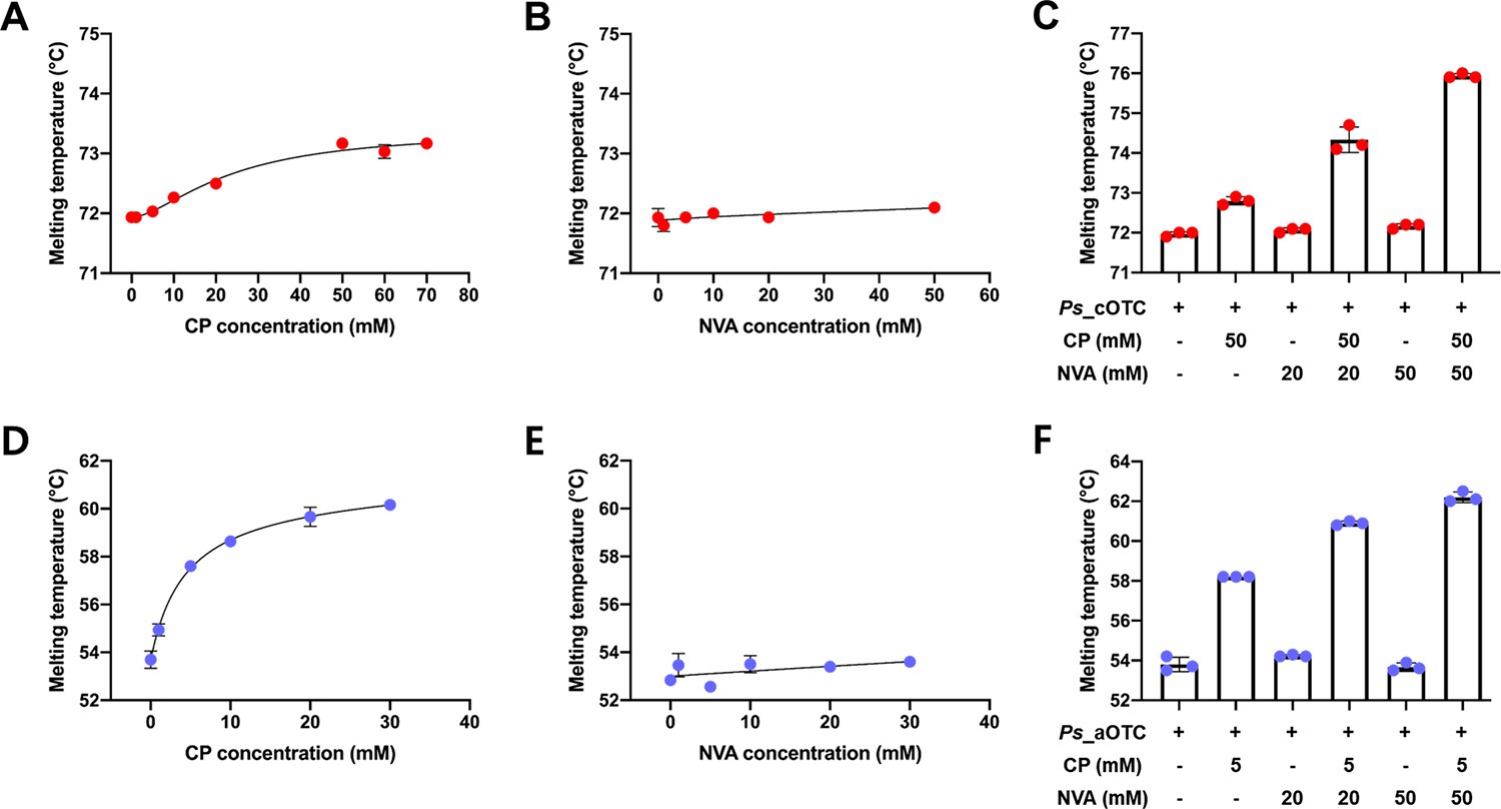
Thermal stability of *Ps*_cOTC (A, B, C) and *Ps*_aOTC (D, E, F) with various concentration of CP and NVA. The stability of OTC was measured in 20 mM Tris-HCl (pH = 8.0) after 15 min of incubation at 295K. The melting temperature is determined by the inflection point of the curve. The data are representative of three individual experiments.

To understand the biochemical features of OTCs, we applied a label-free thermal stability assay to *Ps*_cOTC and *Ps*_aOTC, which allowed us to study protein unfolding as well as protein-ligand interactions (PLI) and observed protein stabilization upon addition of CP and NVA, a homologue of ornithine as well as a competitive inhibitor of OTCs [18–20]. The thermal stability profile of *Ps*_cOTC indicated that the melting temperature of apo *Ps*_cOTC was 71.9 ± 0.0°C, and the temperature was gradually shifted higher with increasing CP concentration (ΔT = 1.3°C). CP did not show a stabilization effect until 5 mM. However, the melting temperature was increased after 10 mM of CP and saturated at 50 mM of CP, thus indicating that *Ps*_cOTC has a low affinity for CP at low concentrations, but the affinity can be increased at higher CP concentrations with a sigmoidal pattern. *Ps*_aOTC also exhibited thermal stability in concert with elevated CP concentrations but with a hyperbolic pattern. The melting temperature was increased exceedingly even with 1 mM of CP and saturated at 20 mM, whereas CP may be fully occupied in the OTCs. The melting temperature was shifted from 53.7 ± 0.4 to 60.2 ± 0.1°C (ΔT = 6.5°C) (Fig 2A and 2D).

Moreover, we also tested the affinity of NVA to the OTCs by monitoring thermal stability. The data revealed that NVA did not affect the thermal stability of either of the OTCs as a single substrate, indicating a low affinity (Fig 2B and 2E). However, the addition of NVA with 50 mM CP for *Ps_*cOTC and 5 mM for *Ps_*aOTC, concentrations high enough for the CP occupancy of the OTCs, showed NVA concentration-dependent thermal stability, thus demonstrating that CP is a necessary leading substrate and that, following CP binding, NVA can bind to *Ps_*cOTC and *Ps_*aOTC (Fig 2C and 2F). Considering that NVA has a similar structure to ORN, this sequential binding can be applied to CP and ORN. Conclusively, despite indirect measurement, the thermal stability data suggest that *Ps*_cOTC displays cooperative binding of CP with low affinity, whereas *Ps*_aOTC has a high affinity for CP, which may explain the unidirectional catalytic activity. Moreover, the sequential binding of CP and NVA implies that CP binding probably induces conformational transitions that are compatible with ORN binding [2–5].

### Overall architecture of *Ps_*cOTC and *Ps_*aOTC

**Fig 3:**
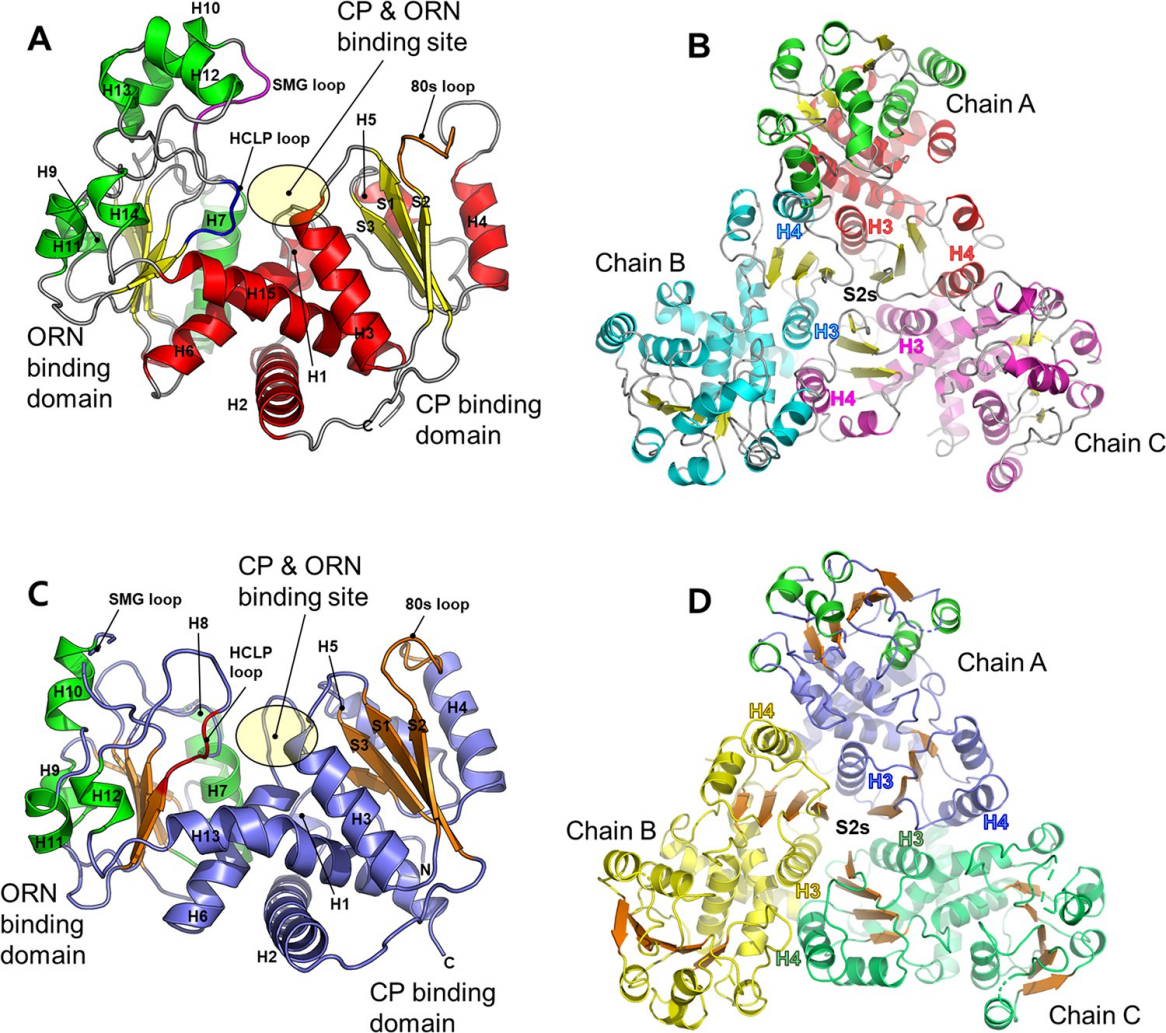
Monomeric and oligomeric structures of *Ps*_cOTC and *Ps*_aOTC from *Psychrobacter* sp. PAMC 21119. (A, B) Monomeric *Ps*_cOTC (red, A) and its trimer (cyan and magenta, B) are shown in cartoon representation. (C, D) Monomeric *Ps*_aOTC (purple-blue, C) and its trimer (yellow and green, D) are also shown in cartoon representation. The strands are yellow for the *Ps*_cOTC and brown for the *Ps*_aOTC. The CP and ORN binding sites are highlighted by yellow circles at the two-domain domain.

To understand the relationship between the structure and function of OTCs, we determined the crystal structures of *Ps*_cOTC and *Ps*_aOTC. The topology and overall structure of *Ps_*cOTC are presented in Fig 3A and 3B. As shown for other OTCs, the *Ps_*cOTC monomer consists of a CP binding domain (residues 1–152) and an ornithine binding domain (residues 153–313). The N-terminal CP binding domain is composed of three parallel β strands (S1 –S4) and surrounded by the amphipathic helices (H3–H5) are. The C-terminal ORN binding domain also contains five parallel β strands (S5–S9) in the domain center and is surrounded by helices (H7–H14). Helix 6 (H6) and the last helix (H15) act as a bridge located in the interface of two domains. H2 is located under H6 and H15. The crystal structure of *Ps*_cOTC revealed that it exists as a trimer in the asymmetric unit, as shown in Fig 3B. The CP binding domains are responsible for the formation of the trimer (see the next sub section “Oligomeric state of OTCs”). The *Ps_*cOTC forms a concave and funnel-like active site located at the interface of the two domains. The CP binding site sits on the loop between S1 and H3, where the conserved STRTR motif is clearly defined. The ORN binding site is proximate to the CP binding site and surrounded by the HCLP loop, the SMG loop, the edge loop of H7, and the loop between S4 and H6 (Fig 3A).

**Fig 4:**
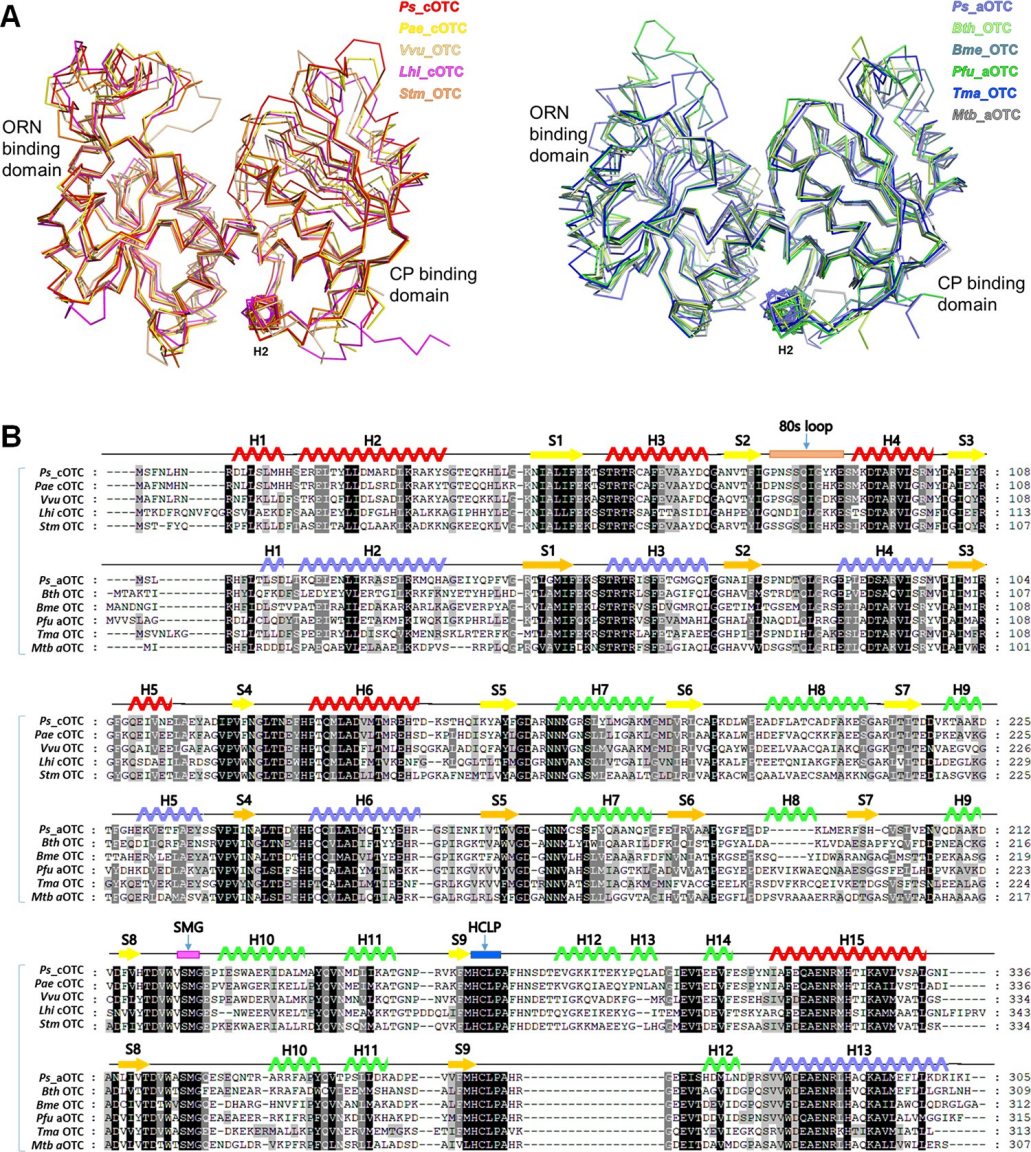
Structural comparison and multiple sequence alignment of *Ps*_cOTC and *Ps*_aOTC with orthologues. (A) Least-squares superposition of OTCs with *Ps*_cOTC and Ps_aOTC. Ribbon representation with different color code was applied for each protein. (B) Multiple sequence alignment of *Ps_*cOTC and *Ps_*aOTC with their orthologs. The alignment was generated using Clustal X [24]. After alignment, the two groups were separated, and secondary structure elements are shown with reference to *Ps_*cOTC and *Ps_*aOTC on the top of the alignment. Conservation of residues of 100%, 80%, and 60% are colored black, grey, and white, respectively. The organism abbreviations are defined as: *Pae*, *Pseudomonas aeruginosa*; *Vvu*, *Vibrio vulnificus*; *Lhi*, *Lentilactobacillus hilgardii*; *Stm*, *Salmonella typhimurium*; *Bth*, *Burkholderia thailandensis*; *Bme*, *Brucella melitensis*; *Pfu*, *Pyrococcus furiosus*; *Tma*, *Thermotoga maritima; Mtb*, *Mycobacterium tuberculosis*.

Moreover, we determined the crystal structure of *Ps_*aOTC at a 2.2 Å resolution. Most of the amino acids were built, except for the SMG loop region (nine amino acids) between S8 and H10 because of ambiguous electron density. The monomer comprises of N-terminal CP binding and C-terminal ORN binding domains. The two domains are connected by helices H6 and H13, which are located at the interface of the two domains, with a crossing formation. Each domain is based on a pleated β-sheet with parallel strands encircled by helices (Fig 3C and 3D). Similar to that of *Ps_*cOTC, the active site of *Ps_*aOTC is located in a pocket between the domains. The bottom of the active site consists of H3, H6, and H13, while the entrance region of the active site is surrounded by the HCLP loop between S9 and H12, the SMG loop between S8 and H10, and a loop between S5 and H7. In detail, His131 from H6; Asn162 and Met163 from H7; the Cys259, Leu260, and Ala262 from the HCLP loop; and Arg55 from H3 are the main residues creating the potential binding cleft for the CP and ORN of *Ps_*aOTC. These residues are highly conserved among OTCs indicating that *Ps_*aOTC may have the catalytic characteristics of aOTCs (Fig 4B).

When we compared *Ps_*aOTC with orthologues from the DALI server (S4 Table), the overall structures were similar to each other (Fig 4A). Multiple sequence alignment also confirmed that the residues forming the active site, including Cys303 and Asp263 which are considered important residues for the deprotonation of the N^ɛ^ of ORN during the first step of the catalytic mechanism of human aOTC [21] are well conserved among OTCs from different species (Fig 4B).

Three identical *Ps_*aOTC monomers are built from a cyclic homotrimer, which shows a similar quaternary structure as *Ps_*cOTC. The homologue search indicated that *Ps_*aOTC has a similar tertiary structure to *Bth* aOTC (PDB: 4F2G, root mean square deviation (RMSD): 2.04 Å) [22], *Bme* OTC (PDB: 4OH7, RMSD: 1.5 Å), *Pfu* OTC (PDB: 1PVV, RMSD: 2.50 Å) [23], *Tma* OTC (PDB: 1VLV, RMSD: 2.04 Å), and *Mtb a*OTC (PDB: 2P2G, RMSD: 2.03 Å) [17] (Fig 4) (S4 Table).

### Oligomeric state of OTCs

Diverse oligomeric states have been identified in OTC orthologues. For instance, the *Pae* cOTC (PDB code: 1ORT) is a dodecamer [8], the *Lhi*_cOTC (PDB code 2W37) [9] is a hexamer, and the cOTC (PDB code 3GRF) from *Giardia lamblia* [25] and the *E*.*coli*_aOTC [26] are trimers in solution. To examine the oligomeric state of *Ps_*cOTC and *Ps_*aOTC, we performed size-exclusion chromatography (SEC). The SEC analysis indicated that *Ps_*cOTC and *Ps_*aOTC are 121.3 kDa and 106.7 kDa, respectively, corresponding to the formation of a trimeric structure in solution (theoretical MW: 38.3 kDa for *Ps_*cOTC and 34.9 kDa for *Ps_*aOTC including GSH and additional amino acids at the N-terminus from the expression vector) (S1 Fig). However, the generation of symmetry mates using *Ps_*cOTC monomers from an asymmetric unit revealed a dodecameric organization with four homotrimers arranged in a tetrahedral manner, which is identical to the dodecameric state of *Pae* cOTC. In *Pae* cOTC, it was recognized that the four 3-fold symmetry axes at the vertices of the tetrahedron serve as the principal interfaces between the homotrimers, generating a dodecameric structure. *Pae* cOTC consists of basically charged residues at the interface of the 3-fold symmetry axes. In particular, R28 and R32 were specified to represent important residues forming the dodecameric assembly [11, 14]. Similar to the *Pae* cOTC configuration, *Ps*_cOTC also exhibits these residues around the 3-fold symmetry interface while exhibiting a trimer in solution. A previous study showed that OTCs from *M*. *abyssi* exhibit salt concentration-dependent oligomerization [15]. We hypothesize that *Ps_*cOTC has this particular property because the crystallization conditions for *Ps_*cOTC included a high salt concentration. However, oligomerization with varying salt concentrations using SEC indicated that *Ps_*cOTC remained a trimer even at 1 M of NaCl; therefore, the dodecameric assembly could be a crystallization artifact. *Ps_*aOTC exhibited a trimer in the crystallographic assembly and identical SEC outcomes were obtained with high salt concentration, similar to *Ps_*cOTC (S1 Fig).

Owing to the ambiguous observations regarding the oligomerization of the OTCs, analytical ultracentrifugation (AUC) was used as a second layer of evidence to determine the exact molecular weight of the OTCs in solution. The experimentally calculated molecular masses of the *Ps_*cOTC and *Ps_*aOTC were 120.8 kDa and 103.2 kDa, respectively (Table 1 and S2 Fig), confirming that the OTCs are trimer. In both structures, the trimers form with tight interactions in a triangle shape. The main portions linking the trimers were regions from the CP binding domain, ranging from amino acid 45–101 for *Ps_*cOTC and 41–97 for *Ps_*cOTC. In particular, the H4 located outside of the CP binding domain was deeply involved with the H3 from the neighboring chain in parallel (Fig 3B and 3D).

**Table 1:** Molecular weight (M) of *Ps*_cOTC and *Ps*_aOTC determined by analytical ultracentrifugation (AUC).

Protein	*Ps*_cOTC	*Ps*_aOTC
M.W. (kDa) (theoretical)	114.9	104.7
M.W. (kDa) (AUC)	120.8	103.2

### Structural differences between *Ps_*cOTC and *Ps_*aOTC

**Fig 5:**
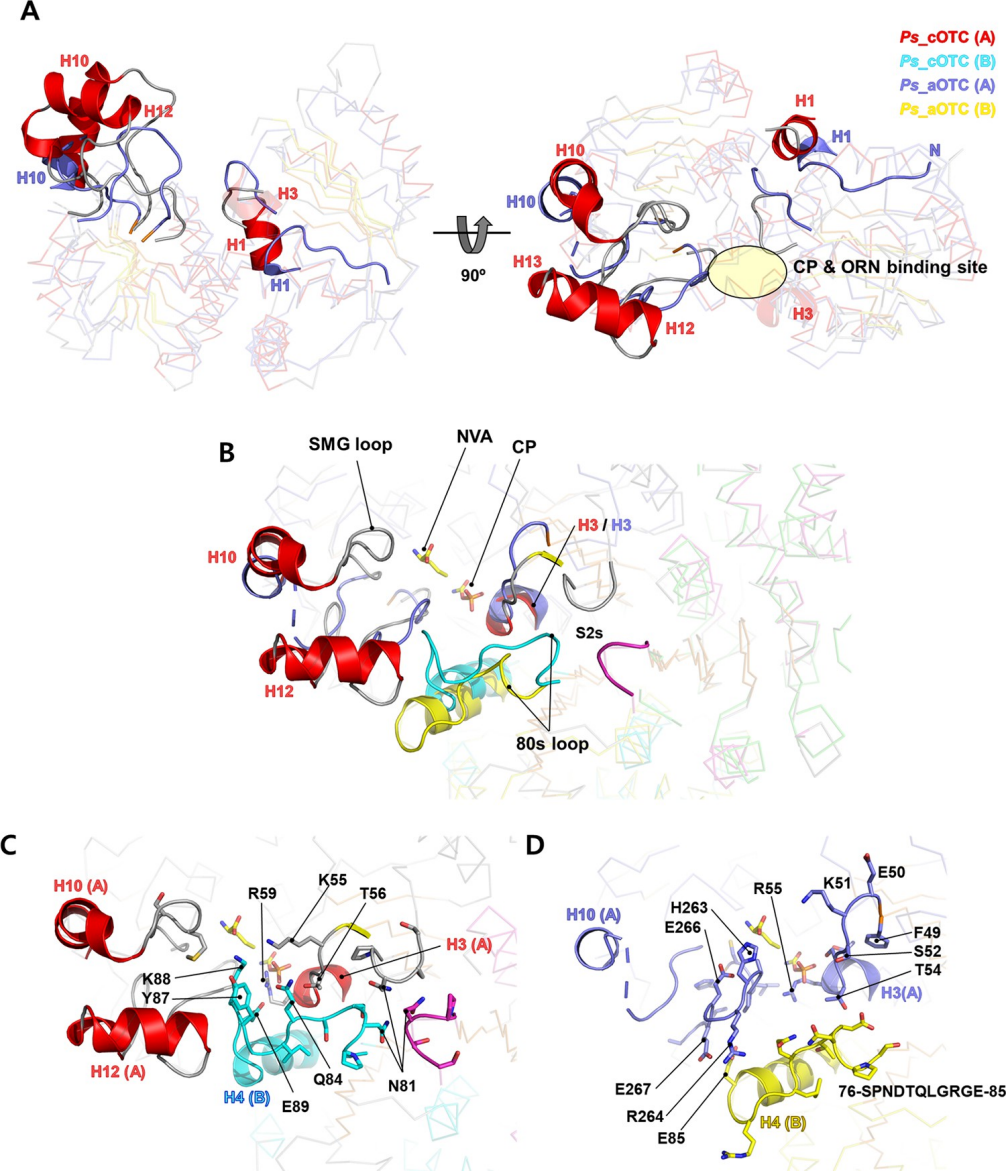
Structural comparison of *Ps*_cOTC and *Ps*_aOTC. (A) Structural superposition between monomeric *Ps*_cOTC (red and grey) and *Ps*_aOTC (purple-blue) are shown in ribbon representation. The different regions between the two OTCs are highlighted with cartoons and thick lines. The catalytic pocket is shown as a yellow circle. (B) Active sites of *Ps*_cOTC and *Ps*_aOTC with neighboring monomers (chain B). The CP and NVA from *V*. *vulnificus* (PDB code: 4H31) are indicated by yellow sticks. Detailed structural features of *Ps*_cOTC (C) and *Ps*_aOTC (D) from Fig 5B with the same orientation. The monomer used is the same as for *Ps*_cOTC and *Ps*_aOTC in Fig 3.

To understand the allosteric effects and substrate preferences of the OTCs, we focused on the structural differences between *Ps_*cOTC and *Ps_*aOTC in the trimer. The *Ps_*cOTC structure has a similar tertiary architecture to that of *Ps_*aOTC as indicated by the 2.45 Å of the RMSD (a total of 242 alpha carbons for the monomer). However, a structural comparison analysis revealed several distinct regions that may underlie crucial aspects of CP cooperativity. When compared with *Ps_*aOTC, *Ps_*cOTC has additional amino acids between S8 and H14, which was reflected in the formation of H12 and H13 in the *Ps_*cOTC structure (Fig 4). Another structural difference around the active site is H10. Unlike *Ps_*aOTC, *Ps_*cOTC forms a long H10 helix extending towards the solvent area and is stabilized via hydrophobic interactions with H12 and H13. V233, V235, I241, W244, F278, I289, Y293, and I300 have been identified as residues involved in this interaction (Fig 5A and 5B and S3 Fig). The formation of H12 and H13 influences the positioning of the 80s loop (85–IGYKES–90) between S2 and H4 of the neighboring monomers, from the *Ps_*cOTC trimer (the chains of the trimer are marked as A, B, and C for both OTCs). In the *Ps_*aOTC structure, the 80s loop (81–LGRGEP–86) is leaning against the H5 of the B chain starting from the sharp turn of P86. However, the corresponding 80s loop of *Ps_*cOTC protrudes into the CP binding site. Notably, the E89 of chain B interacts via a salt bridge with the R59 of chain A (2.7 Å), which is a crucial residue forming the active site for the catalytic reaction. In addition, the 80s loop of *Ps_*cOTC is further stabilized by interacting with the H3 of chain A via a combination of hydrophobic interactions and hydrogen bonds. Interestingly, the C-terminal regions of the 80s loops from the *Ps_*cOTC trimers were gathered to form a tight triangle at the center of the trimer with N81, which was not observed in *Ps_*aOTC. In this arrangement, CP binding may trigger conformational transition in *Ps_*cOTC, and this loop is most likely responsible for the CP-induced allosteric changes in the OTCs observed in the thermal shift activity (Fig 5C and 5D).

**Fig 6:**
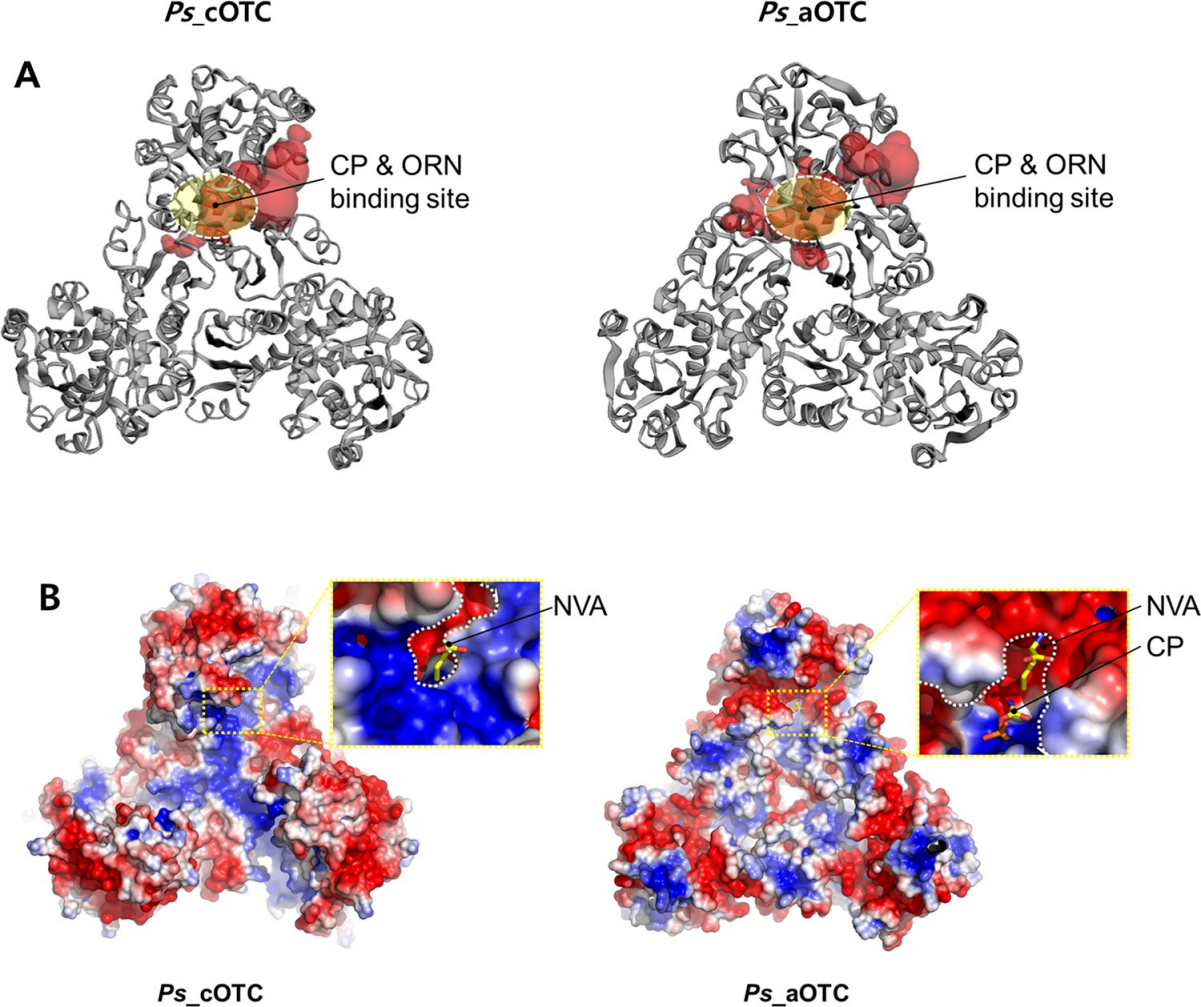
Comparison of the substrate binding site. (A) Measurement of concave surface regions on *Ps*_cOTC and *Ps*_aOTC using the Computed Atlas of Surface Topography of proteins (CASTp). The pocket regions of each protein are visualized with red blobs. The active site is indicated with a white dotted circle. (B) The surface electrostatic potential of OTCs is shown as red (−3 kT/e) and blue (+3 kT/e) surfaces. Closed-up views of each binding pocket with CP and NVA bound to *Vvu* OTC from *Vibrio vulnificus* (PDB code: 4H31) are indicated by yellow sticks.

Another structural difference between the two OTCs is the size of the CP and ORN binding pockets. The dissimilar affinity of OTCs for CP seen in the thermal shift assay suggests that the environment of the active site of *Ps*_cOTC differs from that of *Ps*_aOTC. The *Ps*_cOTC might form a disadvantageous conformation for CP, whereas the *Ps*_aOTC forms a favorable conformation for CP. Consistent with this hypothesis, cavity analysis of the active site indicates that the residues around the active site in the *Ps_*cOTC structure obscure the CP binding site, generating a small binding site (Fig 6A). Superposition of *Ps_*cOTC with *Vvu* OTC complexed with CP and NVA (PDB code: 4H31) showed that M237 from the SMG loop; Q84, Y87, and K88 from the 80s loop of chain B; and K55 between S1 and H3 are the main residues creating a steric barrier that prevents the access of CP to the substrate-binding site. Conversely, *Ps*_aOTC forms a potential binding cavity for CP (Fig 6B). The clear CP and ORN binding sites provided by the lack of closed residues from the 80s loop in the *Ps*_aOTC probably allow the CP molecule to slip into the binding site. These analyses indicate that the 80s loop might be responsible for the CP cooperativity and unidirectional catalysis process of the OTCs. Although a biochemical study and further investigation of structural modifications using *Ps*_cOTC and *Ps*_aOTC might be required to support our hypothesis, our findings suggest that the unique arrangement of the 80s loop, with additional or a lack of residues, generates the different substrate preferences and cooperation activity of the OTCs.

## Conclusion

**Fig 7:**
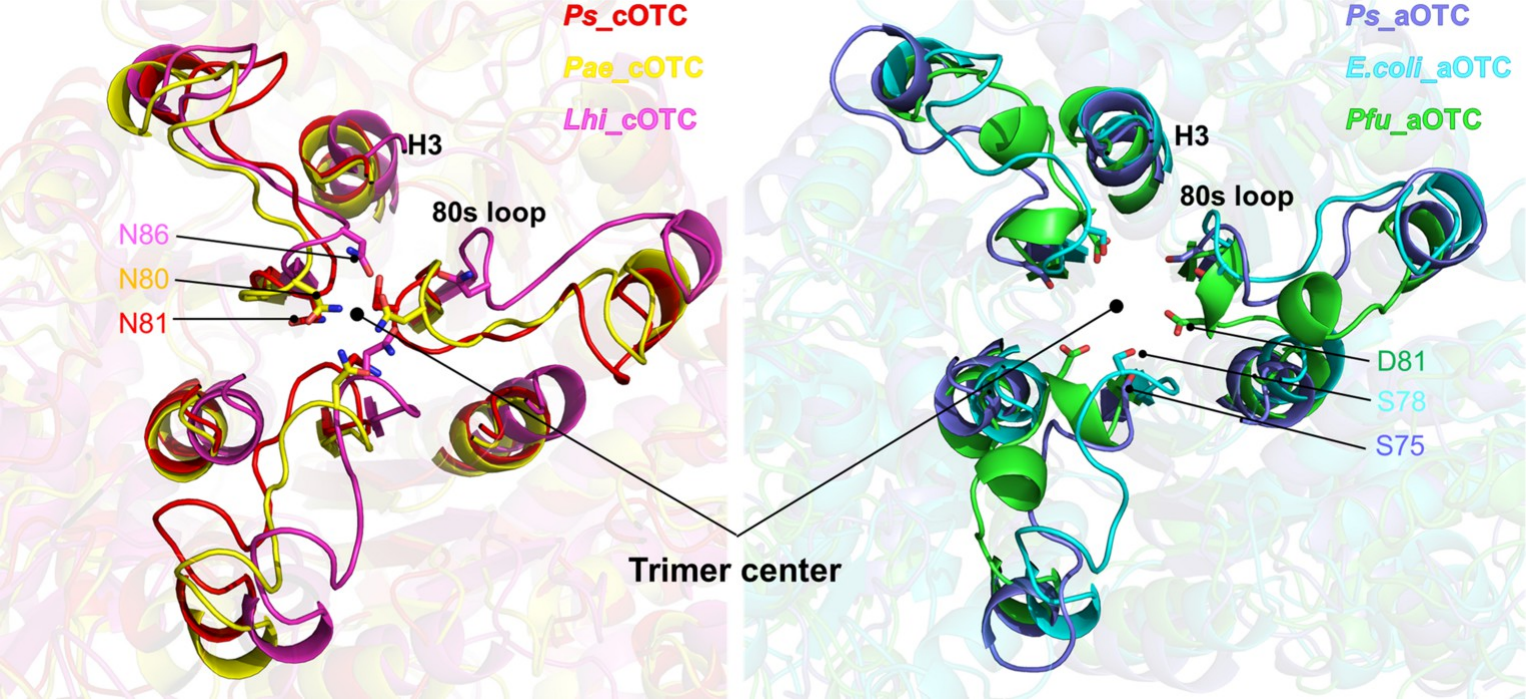
Superposition of the trimer of OTCs. The trimer structures of each orthologue from BLAST were superposed against *Ps*_cOTC and *Ps*_aOTC for each group. The H3 and 80s loops around the trimer center of each orthologue were highlighted with different colors. The closest residue to the trimer center from each OTC is indicated by sticks.

The structural characteristics of OTCs from various species have been deposited in a database and discussed. Previous studies have shown that *Pae* cOTC, the closest structure to *Ps*_cOTC, exhibited homotropic CP cooperativity with little consequence with dodecameric state [12], whereas aOTC did not show any CP cooperativity [1]. This indicates that conformation changes at the contact site among chains can be induced upon CP binding to cOTC but not to aOTC. Initially, the E105 of *Pae* cOTC was recognized as the critical residue for homeotropic cooperativity because E105A and E105G mutants lost cooperativity. However, mutating the residue corresponding E105 to glutamate in the *E*.*coli* aOTC failed to gain cooperativity [3]. This indicates that other factors distinguish the CP cooperativity between two OTCs. Structural comparison of *Ps_*cOTC and *Ps_*aOTC indicates that the 80s loop might be responsible for their cooperativity, owing to its location proximal to the interface of the monomers forming trimers and connecting the CP binding site to the central triangle region. Structure analysis with orthologues also indicated that other cOTCs have *Ps_*cOTC-like configurations within the 80s loop, whereas aOTC orthologues are different (Fig 7).

Another finding from our study was that the substrate preference of the OTCs, identified using a thermal shift assay, can elucidate the unidirectional catalytic activity. Previous studies have shown limited information on this particular protein because of the difficulty of generating the system and activity measurement *in vivo*. Here we showed that the thermal shift assay is an applicable method for assessing the substrate binding of OTCs. The thermal shift assay with various ligands indicated that the *Ps*_cOTC has a low affinity for CP and NVA, whereas *Ps*_aOTC has a high affinity for CP. Moreover, sequential binding of CP and NVA suggests that the *Ps*_cOTC and *Ps*_aOTC undergo structural changes upon CP binding. The conformation transition of *Ps*_aOTC might be higher than that of *Ps*_cOTC as indicated by the substantial increment in the melting temperature of *Ps*_aOTC upon CP binding. These conclusions still need to be validated with the substrate affinity to identify the precise catalytic mechanism; however, our structural and biochemical characterization, comparing cOTC and aOTC from the same species provides new insights into the unidirectional catalysis of OTCs and their cooperative conduct, which are distinguishable features of these two metabolically specialized proteins.

## Materials and methods

### Protein clustering

The remote homologs were searched using ProtBLAST/PSI-BLAST [27]. The sequence of *Ps_*cOTC and *Ps_*aOTC were BLAST-searched against the Protein Data Bank (PDB) database [28], then the results (E-value cutoff for reporting = 1e^-10^) were reloaded for a second BLAST analysis against the Uniprot sport database. The resulted data with full-length sequences were sent to CLANS [29] for sequence similarity grouping. Shorter and darker connecting lines were used for better sequence similarity.

### Cloning, overexpression, and purification

The *arcB* gene from the genomic DNA of *Psychrobacter* sp. PAMC 21119 was amplified using PCR and the primers *Ps_*cOTC F (5’-AGCAGCGGCCTGGTGCCGCGCGGCAGCCATATGATGAGTTTCAATCTTCACAACCGTG-3’) and *Ps_*cOTC R (5’-CATTTGCTGTCCACCAGTCATGCTAGCCATATTAGATATTACCAAGCGCAGAGACC-3’). It was cloned into the pET-28a plasmid (Novagen, Madison, WI, USA) at the NdeI restriction enzyme site using the LIG cloning method, which generated *Ps_*cOTC with six histidine residues at the N-terminus. *E*. *coli* Trans5a and BL21 (DE3) cells were transformed with the cloned plasmid for plasmid storage and recombinant protein expression, respectively. Similarly, the *argF* gene was amplified using primers *Ps_*aOTC F (5’-AGCAGCGGCCTGGTGCCGCGCGGCAGCCATATG ATGAGTTTGCGCCATTTTTTAACC-3’) and *Ps_*aOTC R (5’- CATTTGCTGTCCACCAGTCATGCTAGCCATATTAAATTTTGATCTTATCTTTTAGC-3’) and cloned into the pET-28a for *Ps_*aOTC expression.

The *E*. *coli* strain BL21 (DE3) containing the cloned plasmid was grown in Luria–Bertani broth (Fisher Scientific, Cleveland, OH, USA) supplemented with kanamycin at 50 μg/mL until an optical density of 0.5 at 600 nm was achieved. The expression of recombinant OTCs was induced by adding 1.0 mM isopropyl β-D-1-thiogalactopyranoside. The OTCs were purified in two steps: his-tagged purification using Ni^2+^ affinity chromatography (Qiagen, Hilden, Germany) and FPLC using a Superdex 200 prep grade column (GE Healthcare, Piscataway, NJ, USA). The purity of OTCs was determined by densitometry using Coomassie brilliant blue-stained SDS-PAGE gel (~95%). The purified OTCs were concentrated to 10 mg/mL (0.26 mM) for *Ps*_aOTC and 20 mg/mL (0.52 mM) for *Ps*_cOTC in buffer (20 mM Tris-HCl pH 8.0 and 200 mM NaCl, flash-frozen in liquid nitrogen and stored at −80°C prior to use).

### Thermal shift assay

Label-free thermal shift assay experiments examining OTCs were performed using a Tycho NT.6 instrument (NanoTemper Technologies GmbH, Munich, Germany). Protein samples were diluted (1 mg/mL) in buffer (20 mM Tris HCl pH 8.0) and incubated for 15 min with various substrate concentrations. After 15 min of incubation, the samples were heated in a glass capillary from 35°C to 95°C at a rate of 30°C/min. The intrinsic fluorescence from tryptophan and tyrosine residues was recorded at 330 nm and 350 nm. The ratio of fluorescence (350/330 nm) and the melting temperature was calculated using the internal evaluation features of the Tycho instrument. The data represent the results from three independent experiments.

### Size exclusion chromatography

Size exclusion chromatography for the molecular weight was performed to identify the oligomerization state of the OTCs. The chromatography assay was carried out using a Superdex 200 10/300 GL column equilibrated with 20 mM Tris-HCl (pH 8.0) and NaCl concentrations of 300 mM, 500 mM, and 1,000 mM (column volume 24 mL). A protein sample was diluted (1 mg/mL) in the same buffer then applied to the column and eluted at a flow rate of 0.4 mL/min. The column was calibrated with the Ribonuclease A (13.7 kDa), ovalbumin (44.3 kDa), gamma globulin (150 kDa), and thymoglobulin (669 kDa) for the standard curve.

### Analytical ultracentrifugation

Sedimentation velocity analytical ultracentrifugation experiments were conducted using a ProteomeLab XL-A (Beckman Coulter, Fullerton, CA, USA) analytical ultracentrifuge configured with a scanning UV/Vis detection system. A total of 0.4 mL of protein samples and 0.42 mL of a reference buffer (20 mM Tris-HCl, pH 8.0, 200 mM NaCl) were loaded. The samples were analyzed at 20°C and 42,000 RPM and scanned every 6 min until complete sedimentation was achieved. The sedimentation profile was monitored at 280 nm, and the data were analyzed using SEDFIT software.

### Crystallization and data collection

The crystallization of *Ps_*cOTC was performed using the hanging-drop vapor diffusion method under the following conditions: 0.1 M Tris-HCl, pH 8.5, and 1.5 M lithium. This condition was further optimized and diffraction quality protein crystals were obtained under conditions of 0.1 M Tris-HCl, pH 8.1, and 1.3 M lithium sulfate. X-ray diffraction data sets were collected on the 5C beamline at Pohang Light Source (PLS) operated by the Pohang Accelerator Laboratory (PAL) using perfluoropolyether as a cryo-protectant [30]. For the crystallization of *Ps_*aOTC, *Ps_*aOTC crystals of decent size and quality were grown in 0.1 M sodium acetate:HCl, pH 4.6, and 1 M ammonium citrate dibasic. Full X-ray diffraction data sets were collected using a single *Ps_*aOTC crystal briefly soaked in 30% PEG3350. XDS [31], XSCALE, and MOLREP integrated with CCP4 [32] were used for data reduction, scaling, and phasing.

### Structure determination and refinement

The *Ps_*cOTC and *Ps_*aOTC structures were determined at a 2.6 Å and 2.2 Å resolution using the structure of full-length *Pae* cOTC from *P*. *aeruginosa* (PDB: 1ORT) for *Ps_*cOTC and *Bth* aOTC from *B*. *thailandensis* (PDB: 4F2G) for *Ps_*aOTC as search models without water molecules using the molecular replace method. As the asymmetric unit contains multiple copies of the molecules, local non-crystallographic symmetry restraints were utilized during the refinement [33]. A simple flat bulk solvent model implemented in the program REFMAC was applied for bulk solvent correction [34], and 5% of the reflections were selected for the Rfree calculations [35]. After restrained refinement with REFMAC5 [36], Phenix.refine [37], and a manual process employed using COOT [38], the structures were refined with excellent stereochemistry and geometry.Detailed statistics for data processing and refinement are shown in S2 Table. All figures were produced using Pymol [39].

### Protein Data Bank accession numbers

The final refined coordinates and structure factors of *Ps_*cOTC and *Ps_*aOTC have been deposited in the RCSB Protein Data Bank with accession codes 7XJT and 7X99, respectively.

## Supporting information

S1 Table Pairwise sequence identity of OTCs. (PDF)

S2 Table X-ray diffraction data collection and refinement statistics. (PDF)

S3 Table Structural homologue search results for *Ps*_cOTC from a DALI search (DALI-Lite server). (PDF)

S4 Table Structural homologue search results for *Ps*_aOTC from a DALI search (DALI-Lite server). (PDF)

S1 Fig Size exclusion chromatography analysis of purified OTCs with various NaCl concentrations. (PDF)

S2 Fig Sedimentation velocity analytical ultracentrifugation of *Ps_*cOTC. (PDF)

S3 Fig Stereo view of superposition showing the H10, H12, and H13 regions of *Ps*_cOTC (red and cyan) and the H10 region of *Ps*_aOTC (blue). (PDF)

## Abbreviations

OTC: ornithine carbamoyltransferase
ORN: ornithine
CP: carbamoyl phosphate
ADI: arginine deiminase
NVA: l-norvaline
PDB: protein data bank
RMSD: root mean square deviation
IPTG: isopropyl β-D-1-thiogalactopyranoside
AUC: analytical ultracentrifugation
