# Supplementary material for: Comparative structural insight into the unidirectional catalysis of ornithine carbamoyltransferases from Psychrobacter sp. PAMC 21119
Source: PLoS One. 2022 Sep 23;17(9):e0274019. doi: 10.1371/journal.pone.0274019 (PMC9506655; doi:10.1371/journal.pone.0274019)
Supplement: S1 Table — (PDF) [file pone.0274019.s001.pdf]

S1 Table. Pairwise sequence identity of OTCs. The percentage identity and similarity of each pair of amino acid sequences is shown at the upper and lower half of each box, respectively. Web-based EMBOSS Needle [1] was used for pairwise alignment. The default settings for the gap and the extended penalty were 10 and 0.5, respectively.

	Ps_cOTC	*Pae*_OTC	*Ps_aOTC*	*Q4FT48*[*]
Ps_cOTC	100<br>100			
Pae_OTC	77.7<br>89.3	100<br>100		
Ps_aOTC	32.4<br>52.2	32.9<br>51.8	100<br>100	
*Q4FT48**	32.5<br>52.5	33.0<br>52.3	95.8<br>98.4	100<br>100

*OTC from *Psychrobacter arcticus* (strain DSM 17307)

1. Needleman S.B. and Wunsch C.D. A general method applicable to the search for similarities in the amino acid sequence of two proteins. J. Mol. Biol. ;1970 48(3):443-5
