# Supplementary material for: Comparative structural insight into the unidirectional catalysis of ornithine carbamoyltransferases from Psychrobacter sp. PAMC 21119
Source: PLoS One. 2022 Sep 23;17(9):e0274019. doi: 10.1371/journal.pone.0274019 (PMC9506655; doi:10.1371/journal.pone.0274019)
Supplement: S2 Table — (PDF) [file pone.0274019.s002.pdf]

S2 Table. X-ray diffraction data collection and refinement statistics.

**Dataset**	***Ps*_cOTC**	***Ps*_aOTC**
X-ray source	PLS BL-5C beamline	PLS BL-7A beamline
Space group	R3	$P6_3$
Unit-cell parameters (Å, °)	a = b = 130.06, c = 329.32, α = β = 90.0, γ = 120.0	a = b = 129.61, c = 75.20, α = β = 90.0, γ = 120.0
Wavelength (Å)	0.9794	0.97949
Resolution (Å)	29.40–2.60 (2.65–2.6)	29.76–2.20 (2.27–2.20)
Total reflections	338,708	763,950
Average I/σ (I)	31.3 (2.8)	25.9 (2.7)
R_{merge}[a]	0.077 (0.563)	0.075 (1.326)
CC1/2	0.995 (0.959)	1.000 (0.824)
Redundancy	5.3 (5.5)	20.9 (21.8)
Completeness (%)	99.9 (99.7)	100.0 (100.0)
**Refinement**		
Resolution range (Å)	46.53–2.60 (2.68–2.60)	29.78–2.20 (2.26–2.20)
No. of reflections of working set	60,425 (4,472)	34,775 (2,521)
No. of reflections of test set	3,460 (206)	1,852 (159)
No. of amino acid residues	1347	588
Number of conformers	4	2
R_{cryst}[b]	0.20 (0.34)	0.18 (0.25)
R_{free}[c]	0.23 (0.33)	0.23 (0.33)
R.m.s. bond length (Å)	0.007	0.009
R.m.s. bond angle (°)	1.000	1.588
Average B value ($Å^2$) (protein)	80.27	55.38
Average B value ($Å^2$) (solvent)	149.03	48.81
Ramachandran favored (%)	94.40	96.02
Ramachandran allowed (%)	4.37	3.63
Ramachandran outliers (%)	1.23	0.35

[a] $R_{merge} = \sum | \langle I \rangle - I | / \sum \langle I \rangle$.

[b] $R_{cryst} = \sum | |Fo| - |Fc| | / \sum |Fo|$.

[c] R_{free} calculated with 5% of all reflections excluded from the refinement stages using high-resolution data.

Values in parentheses refer to the highest resolution shells.
