# Supplementary material for: Comparative structural insight into the unidirectional catalysis of ornithine carbamoyltransferases from Psychrobacter sp. PAMC 21119
Source: PLoS One. 2022 Sep 23;17(9):e0274019. doi: 10.1371/journal.pone.0274019 (PMC9506655; doi:10.1371/journal.pone.0274019)
Supplement: S3 Table — (PDF) [file pone.0274019.s003.pdf]

S3 Table. Structural homologue search results for *Ps*_cOTC from a DALI search (DALI-Lite server)

Protein	PDB code	DALI Z-score	UniProtKB code	Sequence % I.D. with *Ps*_cOTC (aligned residue number)	Reference
Pae cOTC (*Pseudomonas aeruginosa*)	1ORT	43.7	P08308	76 (324/335)	[1]
Vvu OTC (*Vibrio vulnificus*)	4JFR	40.9	Q8DCF5	61 (318/334)	CSGID
Lhi cOTC (*Lactobacillus hilgardii*)	2W37	39.2	Q8G998	49 (317/341)	[2]
*Stm*_OTC (*Salmonella typhimurium)*	6OVW	38.3	A0A0H3NPU3	57 (319/334)	CSGID

*CSGID, Center for Structural Genomics of Infectious Diseases; N.D., not determined.

1. Villeret V, Tricot C, Stalon V, Dideberg O. Crystal structure of Pseudomonas aeruginosa catabolic ornithine transcarbamoylase at 3.0-A resolution: a different oligomeric organization in the transcarbamoylase family. Proc Natl Acad Sci. 1995;92(23): 10762–6.

2. de las Rivas B, Fox GC, Angulo I, Ripoll MM, Rodríguez H, Muñoz R, et al. Crystal Structure of the Hexameric Catabolic Ornithine Transcarbamylase from Lactobacillus hilgardii: Structural Insights into the Oligomeric Assembly and Metal Binding. J Mol Biol. 2009;393(2): 425–34.
