# Supplementary material for: Comparative structural insight into the unidirectional catalysis of ornithine carbamoyltransferases from Psychrobacter sp. PAMC 21119
Source: PLoS One. 2022 Sep 23;17(9):e0274019. doi: 10.1371/journal.pone.0274019 (PMC9506655; doi:10.1371/journal.pone.0274019)
Supplement: S4 Table — (PDF) [file pone.0274019.s004.pdf]

S4 Table. Structural homologue search results for *Ps*_aOTC from a DALI search (DALI-Lite server)

Protein	PDB code	DALI Z-score	UniProtKB code	Sequence % I.D. with *Ps*_aOTC (aligned residue number)	Reference
Bth aOTC *(Burkholderia thailandensis)*	4F2G	37.9	Q2T0L1	51 (283/290)	[1]
Bme OTC *(Brucella melitensis)*	4OH7	36.7	C0RH19	47 (285/302)	SSGCID
Pfu OTC *(Pyrococcus furiosus)*	1PVV	36.1	Q51742	45 (285/313)	[2]
Tma OTC *(Thermotoga maritima)*	1VLV	35.6	P96108	41 (283/308)	JCSG
Mtb aOTC *(Mycobacterium tuberculosis)*	2P2G	35.1	P9WIT9	43 (282/308)	[3]

*SSGCID, Seattle Structural Genomics Center for Infectious Disease; JCSG, Joint Center for Structural Genomics; N.D., not determined.

1. Baugh L, Gallagher LA, Patrapuvich R, Clifton MC, Gardberg AS, Edwards TE, et al. Combining Functional and Structural Genomics to Sample the Essential Burkholderia Structome. de Crécy-Lagard V, editor. PLoS One. 2013;8(1): e53851.

2. Villeret V, Clantin B, Tricot C, Legrain C, Roovers M, Stalon V, et al. The crystal structure of Pyrococcus furiosus ornithine carbamoyltransferase reveals a key role for oligomerization in enzyme stability at extremely high temperatures. Proc Natl Acad Sci. 1998;95(6): 2801–6.

3. Sankaranarayanan R, Cherney MM, Cherney LT, Garen CR, Moradian F, James MNG. The Crystal Structures of Ornithine Carbamoyltransferase from Mycobacterium tuberculosis and Its Ternary Complex with Carbamoyl Phosphate and l-Norvaline Reveal the Enzyme's Catalytic Mechanism. J Mol Biol. 2008;375(4): 1052–63.
