# Supplementary material for: Comparative structural insight into the unidirectional catalysis of ornithine carbamoyltransferases from Psychrobacter sp. PAMC 21119
Source: PLoS One. 2022 Sep 23;17(9):e0274019. doi: 10.1371/journal.pone.0274019 (PMC9506655; doi:10.1371/journal.pone.0274019)
Supplement: S1 Fig — (PDF) [file pone.0274019.s005.pdf]

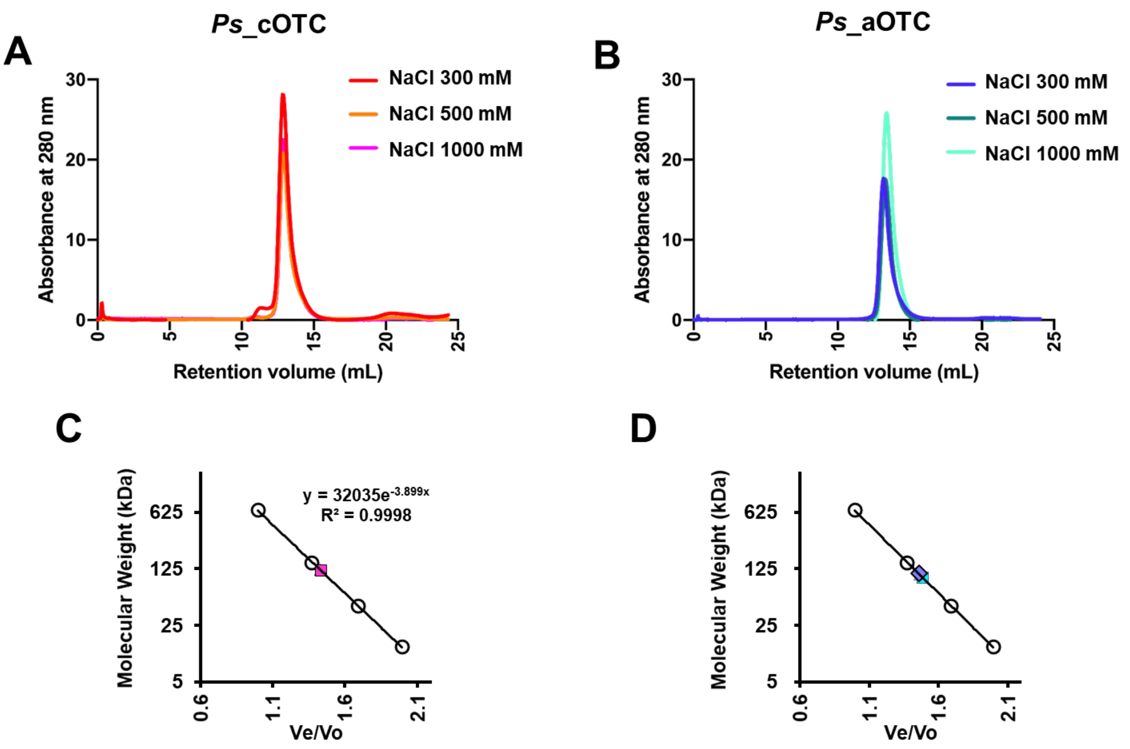

E

	Protein	
NaCl con.	*Ps*_cOTC	*Ps*_aOTC
300 mM	121.338	106.781
500 mM	119.927	100.236
1000 mM	118.481	97.369

S1 Fig. Size exclusion chromatography analysis of purified OTCs with various NaCl concentrations. (A, B) Assessment of OTC oligomerization state in solution using size exclusion chromatography. (C, D) The linear fit of the retention volumes of four protein molecular weight standards (Std), Ribonuclease A (13.7 kDa), ovalbumin (44.3 kDa), gamma globulin (150 kDa), and thymoglobulin (669 kDa) to their log molecular weight is shown as a black line on the graph. The retention volumes of the OTCs with 300 mM, 500 mM, and 1000 mM of NaCl were plotted. (E) The experimental molecular weights of OTCs were calculated based on the retention profile using the standard curve equation.
