# Supplementary material for: Comparative structural insight into the unidirectional catalysis of ornithine carbamoyltransferases from Psychrobacter sp. PAMC 21119
Source: PLoS One. 2022 Sep 23;17(9):e0274019. doi: 10.1371/journal.pone.0274019 (PMC9506655; doi:10.1371/journal.pone.0274019)
Supplement: S2 Fig — (PDF) [file pone.0274019.s006.pdf]

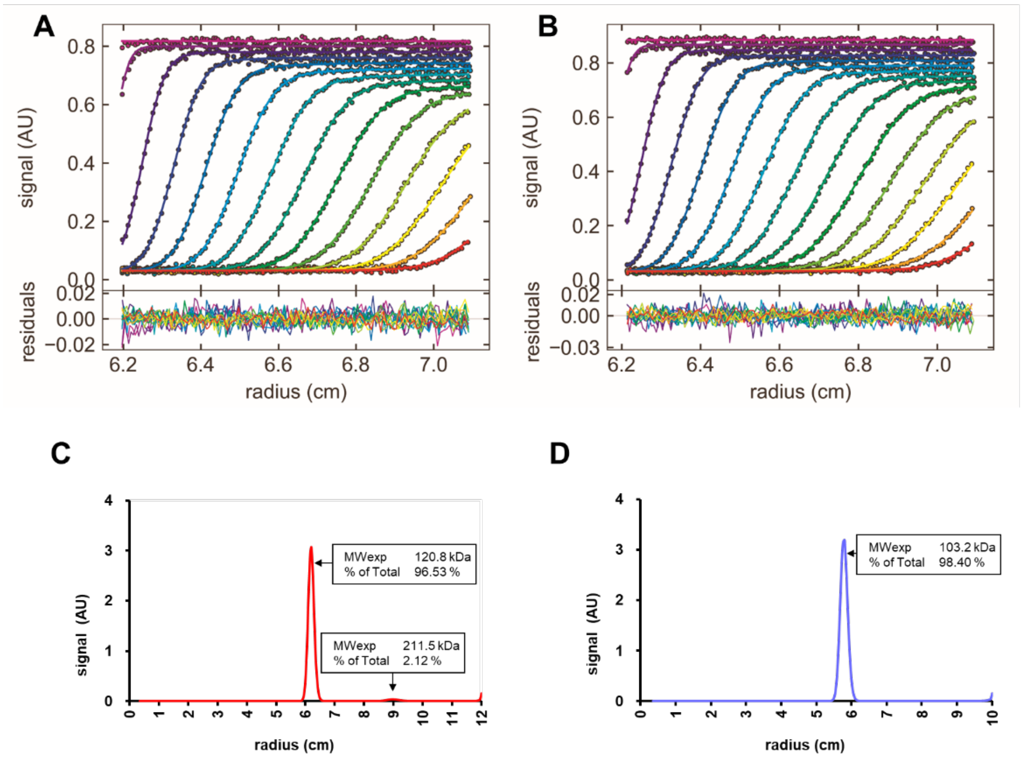

S2 Fig. Sedimentation velocity analytical ultracentrifugation of *Ps*_cOTC. (A, C) and *Ps*_aOTC (B, D). Sedimentation profiles were scanned every 6 min at 280 nm and colored with progressive rainbow colors using the GUSSI program. The parameter from SEDNTERP software (Ver. 3.0.3) was integrated into the SEDFIT software (Ver. 16.36). The experiment was performed in 20 mM Tris-HCl (pH 8.0) and 200 mM NaCl at 20 °C using ProteomeLab XL-A (Beckman Coulter, Inc., Brea, CA, USA).
