# Supplementary material for: Comparative structural insight into the unidirectional catalysis of ornithine carbamoyltransferases from Psychrobacter sp. PAMC 21119
Source: PLoS One. 2022 Sep 23;17(9):e0274019. doi: 10.1371/journal.pone.0274019 (PMC9506655; doi:10.1371/journal.pone.0274019)
Supplement: S3 Fig — (PDF) [file pone.0274019.s007.pdf]

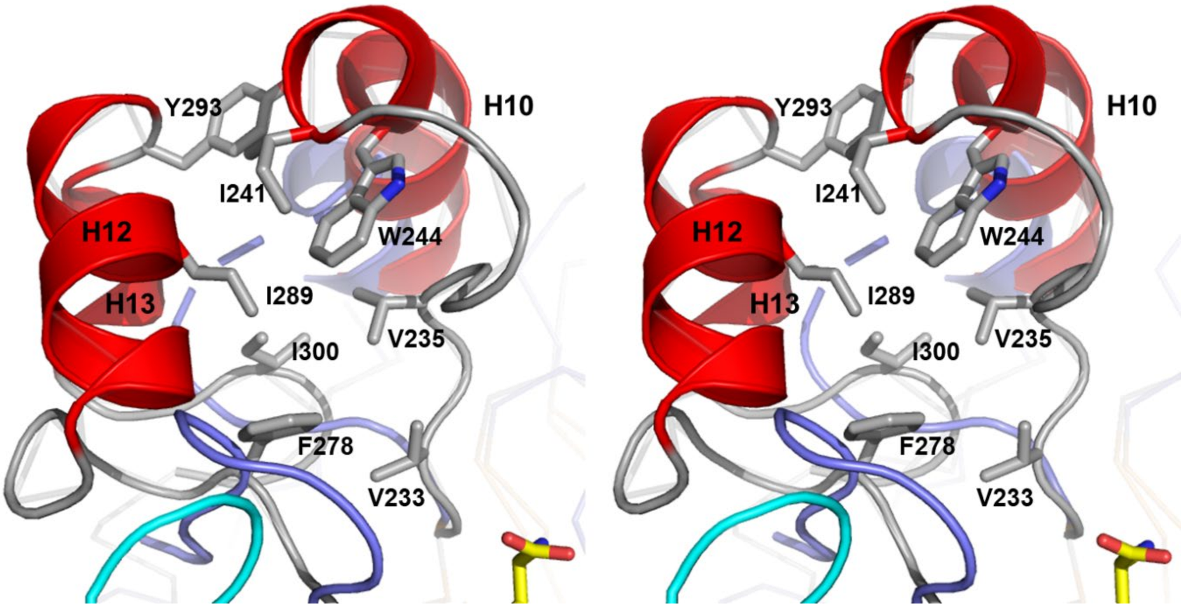

S3 Fig. Stereo view of superposition showing the H10, H12, and H13 regions of *Ps*_cOTC (red and cyan) and the H10 region of *Ps*_aOTC (blue). The residues on the interface of the H10 and H12 of *Ps*_cOTC are shown with sticks.
